# Pre-analytical Practices in the Molecular Diagnostic Tests, A Concise Review

**DOI:** 10.30699/ijp.2020.124315.2357

**Published:** 2020-11-10

**Authors:** Maryam Sotoudeh Anvari, Atoosa Gharib, Maryam Abolhasani, Aileen Azari-Yam, Farzaneh Hossieni Gharalari, Moeinadin Safavi, Ali Zare Mirzaie, Mohammad Vasei

**Affiliations:** 1 *Molecular Pathology and Cytogenetics Division, Pathology Department, Children's Medical Center, Tehran University of Medical Sciences, Tehran, Iran*; 2 *Department of Pathology, Modarres Hospital, Shahid Beheshti University of Medical Sciences, Tehran, Iran*; 3 *Oncopathology Research Center, Iran University of Medical Sciences (IUMS), Tehran, Iran; Hasheminejad Kidney Center, Iran University of Medical Sciences, Tehran, Iran*; 4 *Department of Pathology, School of Medicine, Urmia University of Medical Sciences, Urmia, Iran*; 5 *Department of Pathology, School of Medicine, Iran University of Medical Sciences, Tehran, Iran*; 6 *Cell-based Therapies Research Center, Digestive Disease Research Institute, Shariati Hospital, Tehran University of Medical Sciences, Tehran, Iran *

**Keywords:** Body Fluids, Formaldehyde, Nucleic Acids, Pre-analytical Phase

## Abstract

Molecular assays for detection of nucleic acids in biologic specimens are valuable diagnostic tools supporting clinical diagnoses and therapeutic decisions. Pre-analytical errors, which occur before or during processing of nucleic acid extraction, contribute a significant role in common errors that take place in molecular laboratories. Certain practices in specimen collection, transportation, and storage can affect the integrity of nucleic acids before analysis. Applying best practices in these steps, helps to minimize those errors and leads to better decisions in patient diagnosis and treatment. Widely acceptable recommendations, which are for optimal molecular assays associated with pre-analytic variables, are limited. In this article, we have reviewed most of the important issues in sample handling from bed to bench before starting molecular tests, which can be used in diagnostic as well as research laboratories. We have addressed the most important pre-analytical points in performing molecular analysis in fixed and unfixed solid tissues, whole blood, serum, plasma, as well as most of the body fluids including urine, fecal and bronchial samples, as well as prenatal diagnosis samples.

## Introduction

Nucleic acid-based diagnostic tests are rapidly growing to complement or even replace the traditional diagnostic methods in clinical laboratories. They are also used to predict the outcome and to choose the most effective modality in treating many diseases. They are accurate tests needing very small amounts of DNA or RNA samples to carry out. The results are reproducible but similar to other diagnostic tests, they may have some drawbacks derived from analytic and pre-analytic factors. Some studies have shown that the pre-analytical errors make up to 60-70% of all laboratory errors occurring inside and outside the laboratories (1, 2). Valuable recommendations for standardization of specimen processing in the molecular laboratories are available but there is great variability in specimen handling before delivering the samples to molecular laboratories. This important phase of test performance may lead to false negative or false positive results. Performing reliable procedures in sample processing will increase the accuracy and reproducibility of these diagnostic tests. The major issues of concern in the sample handling are related to nucleic acid integrity, stability, and the effects of some interfering agents during sample transportation, extraction, and storage. 

 Very recently, Compton *et al.* proposed a set of recommendations that are meant to apply to tissue and blood specimens of cancer patients (Compton *et al.*) (3). This article is a report from a group from Precision Medicine Project Team of the CAP (College of American Pathologists) establishing a basic set of evidence-based recommendations for pre-analysis in tissue and blood specimens. Their review was limited to primary data available from published recommendations by ASCO (American Society of Clinical Oncology), Biorepositories and Biospecimen Research Branch, CAP, European Committee for Standardization/Technical Committee, CLSI (Clinical & Laboratory Standards Institute) and some other guidelines. They only focused on pre-analytical factors for either formalin-fixed, paraffin-embedded (FFPE) tissues or blood/plasma biospecimens that impacted analysis data for nucleic acids used for cancer patients. 

 In this article, we have reviewed published papers and recommendations, which have addressed correct practices in specimen collection, transportation, preservation, and storage in most biological samples (including tissue and blood) which are routinely received and processed in pathology as well as translational research laboratories. In addition to considering the cancer biospecimens, the scope of this review was also comprised of common molecular tests to detect many viral and bacterial microorganisms. Most of the articles about pre-analytical studies in human samples were collected from the past 20-year interval. For selecting the articles for citation, we considered the citation rate of the article as well as the rank of the journal which published the article. The descriptive results of that review are presented as below and the summary is depicted in table 1. 

We hope that the implementation of these recommended key pre-analytical procedures in molecular diagnostic laboratories will improve the baseline level of quality for biospecimens and increase the confidence level while reporting the results. 

**Test Selection and Request Form**

Several medical specialists request molecular tests to reach a diagnosis, choose a treatment strategy, or define patients’ prognostic stratification. The clinicians requesting a molecular test should be aware of the power limitations of the requested test in management and decision making as well as the cost-benefit ratio. There are still lots of diseases in which some routine non-molecular tests are more valid than available molecular tests. The physicians should make sure that the request has been decided appropriately with a logical and cost-effective step-wise plan in patient management. Performing necessary but unrequested molecular tests which are done after preliminary diagnoses by pathologists is an accepted strategy within the molecular laboratories (4). In targeted therapy, the exact characterization of gene expression assays and performance of these tests by an in-house pathologist or specialists in laboratory medicine (reflex tests) would be a critical time-saving process. This policy is recommended especially if more than 10% of samples with a given diagnosis, need additional testing. The common examples are *HER2* amplification in breast cancer, *EGFR* mutations and *ALK *rearrangements in lung cancer, *BRAF* mutations in malignant melanoma, *RAS* mutations in colorectal cancer, and *BCR/ABL1* in chronic myelogenous leukemia (4). 

**Recommended Laboratory Practices in Pre-analytical Phase in Different Biological Samples**

**Fixed Tissue Specimens**

Fixation is a fundamental step for long term tissue preservation to prevent autolysis and molecular components decay. Fixed tissue specimens stored in paraffin blocks in pathology departments are frequently used as valuable sources of nucleic acids for molecular assays. Therefore, pre-analytic variables should be taken into consideration to avoid possible misinterpretation of the results.

**1. Types of Fixatives**

The most widely used fixatives include neutral buffered formalin (NBF), ethanol, methanol, Bouin, Carnoy, Zenker, and glutaraldehyde. We briefly mention some of the effects of these fixatives on nucleic acids. Formalin fixation induces DNA-protein and RNA-protein cross-links (5, 6), which may prevent efficient nucleic acid extraction (7) as well as in situ hybridization (8-10). Nucleic acid fragmentation (11) and loss may occur by prolonged fixation of the specimen or changes in the pH of the fixative (12-15). Fixation in un-buffered formalin results in a significant decrease in the quantity of extracted DNA in comparison with buffered formalin and would lead to a less effective DNA detection (16). Formalin fixation can induce random damages in nucleic acids and impress false mutations (17). Postmortem interval and the time between tissue removal from the body (cold ischemia) should be limited to 48 hours and 1 hour respectively, when DNA is analyzed by Fluorescence In Situ Hybridization (FISH), in turn, these thresholds for PCR analysis could be defined as less than 4 days and 24 hours (18). Formalin fixation less than 72 hours is optimal for DNA integrity, although PCR can be done with successful results with longer duration after fixation in formalin and preparation of formalin-fixed paraffin-embedded (FFPE) blocks (18). Some authors recommended that starting formalin fixation in less than 2 hours after removing the tissue from the body, cold fixation (4°C), using cold 10% neutral formalin, fixation time of 3 to 6 hours, adding ethylene-diamine-tetra-acetic acid (EDTA, 20-50 mmol/L), and prevention from low pH environment, all would help in optimization of nucleic acid preservation (12). In comparison to fresh samples, formalin can cause chemical modification of RNA. In an experimental study, despite appropriate RNA integrity scores in FFPE samples, extracted RNA from tissues, exhibited reverse transcription inhibition in quantitative RT-PCR assays especially in samples with long length amplicons. PAXgene fixed samples did not show such inhibition (19). For RNA analysis, the fixation time in NBF should be limited to 48 hours at 4°C or 8 hours at ambient temperature (uncontrolled RT) and the blocks should be analyzed within 1 year (18). Potential influences of many factors such as dehydration, clearing, paraffin reagents, and other conditions on DNA endpoints have remained largely unaddressed in the literature. For more information, we recommend referring to an excellent review written by Bass, B, P *et al.* (18). Despite all these challenges, genomic and gene expression data generated from FFPE specimens often yield acceptable results in comparison to the original fresh samples.

**Ethanol and methanol** are excellent fixatives for preserving both high molecular weight DNA and RNA with little chemical nucleic acid changes (12). In addition to solid tissue preservation, ethanol can be used for transporting and storing tissue at room temperature (RT) (20). Kilpatrick *et al.* suggested that high molecular weight DNA can be efficiently retrieved from tissues stored in ethanol from one day up to 2 years. Although after 6 months, some DNA degradation could be observed in tissues stored in ethanol (20).

**Carnoy’s solution** composed of 60% ethanol, 30% chloroform and 10% glacial acetic acid is one of the best fixatives for optimum maintenance of nucleic acids, especially high-molecular-weight RNA (12, 21). It has been shown that RNAs extracted from epithelial cells and fixed in Carnoy's solution maintain nucleic acid integrity for the beta-actin gene amplified up to 977 base pairs (bps) (22). Carnoy's fixative preserves tissues better than methanol-acetone when examined in more than 3 months post-fixation period (22). Li *et al.* amplified target DNA sequences from cells fixed in Carnoy’s preservative being stored ranging from few weeks up to 6 years at -20°C. They observed no difference between the quality of amplification products from three different sizes of templates (400 bps for retinoblastoma gene, 251 bps for Duchenne muscular dystrophy gene, and 609 bps for the sex-determining region of the Y gene) either extracted from fresh tissues or Carnoy’s-fixed cells (23). 

**Methacarn solution** (60% methanol, 30% chloroform, and 10% glacial acetic acid) has been shown to be an appropriate fixative for preserving tissue RNA regarding both efficiency of extraction and integrity of extracted total RNA. Fragments of RNA with 300 to 700 bps length could be amplified from methacarn-fixed tissues (24). 

**Bouin’s, B5, and Zenker’s solution:** Severe DNA degradation is observed in these fixatives which contain heavy metals. It is not recommended to use mercury-based fixative for nucleic acid isolation. In addition to coagulation of proteins, mercuric acid and chromium cause formation of large metal-nucleoprotein complexes and therefore lower the yield of DNA extraction (25-27). In addition, Bouin’s solution which contains acetic acid and formalin (pH 2) causes de-purination of DNA as well as DNA and RNA damage (25, 28). These RNAs become resistant to RNase and consequently can contaminate DNA extraction products (29). 

**Glutaraldehyde** is a widely used fixative in electron microscopy. It has been shown that high molecular weight (>50 kb) DNA is preserved better in tissues fixed in 0.2 M phosphate-buffered 1% glutaraldehyde solution (at pH 7.0) in comparison with 10% buffered formalin (13). There is no data on RNAs fixed in glutaraldehyde.

**2. Effects of Decalcification**

Commonly used decalcification agents such as nitric acid and hydrochloric acid which contain strong acids, degrade nucleic acids, and decrease their recovery rate from samples (30). The quantity and quality of the extracted nucleic acids are acceptable when decalcification is done using 14% EDTA or formic acid, in comparison to the above mentioned strong acids (30). 

**3. Paraffin**

Paraffin waxes permeate the tissue in liquid form, solidify rapidly when cooled, and preserve tissue structure in sectioning (27). There are variable paraffin waxes regarding quality and melting points (ranging from 47°C to 64°C) (31), which may cause confounding effects on nucleic acid extraction (14, 32). At 60°C, paraffin may cause DNA damage and protein cross-linking (33-35). Paraffin removal is necessary for extracting pure DNA since its remnants cause PCR inhibition (36, 37). Goelz *et al.* first mentioned the inhibitory effect of residual paraffin on DNA yield (38). Chung *et al.* demonstrated that marked deparaffinization is a necessary step for nucleic acid recovery from paraffin-embedded specimens. They demonstrated a 70% reduction in the yield of RNA and amplicon length to less than 300 bps due to formalin fixation and paraffin embedding (33). But another study showed that all (100%) shorter fragments of RNA up to 151 bps were amplified by reverse transcription-polymerase chain reaction (RT-PCR) for housekeeping gene (glucose-6-phosphate dehydrogenase) in paraffin-embedded breast cancer samples (39). However, it should be noticed that with improvements in nucleic acid extraction methods, the confounding effect of paraffin has decreased (40). Although amplification inhibitors may be present in silica membrane-based column extraction and heat treatment methods in single nucleotide polymorphism (SNP) evaluation by real-time, Huijsmans *et al.* recommended using extraction by silica membrane-based column and enhanced magnetic silica. They found that silica membrane-based extraction method and enhancing with magnetic silica could be used for amplification of fragments between 400-600 bps (41). RNA fragmentation in paraffin-embedded tissue samples could happen (42). Páska *et al.* recommended that amplicon up to 225 bps could give good results in gene expression studies (42) but Godfrey *et al.* showed that only RNA amplicons which were less than 130 bps could give acceptable results in RT-PCR (43). For improvement in PCR efficiency, changing some of the thermocycler parameters such as increasing cycle number and duration of each cycle was also helpful (44, 45).

**4. Microtomy**

The optimal thickness of sections in FFPE tissue for nucleic acid evaluation depends on the dimensions and cellularity of the tissues. Generally, 20-µm sections of large tissues or 40 to 80 µm thickness of smaller tissue are recommended for PCR analysis (46). Three 10-µm-thick sections per reaction have been proposed for kidney, prostate, stomach, colon, brain, bone marrow (BM), and lung but for breast tissue, 10 sections with similar thickness is needed in to perform DNA extraction (47). In our experience, five 10-µm thick sections can produce reliable results in nucleic acid quality as well as quantity (unpublished data). Cleaning the blade of microtomes using 100% ethanol (46) and xylene or using a disposable blade (48) are recommended before each block sectioning. For monitoring effective blade cleaning and avoiding carry-over contamination from previous blocks, a blank tissue-free paraffin block should be sectioned (minimum: two sections) between sequential target blocks. The sections of these blanks should undergo DNA extraction steps and be tested in parallel with the samples of interest to ensure no carry-over contamination (46). 

**5. Tissue Conditions**

Although tissue nature is a determining factor, generally 1 to 2 g of tissue would yield satisfactory material for molecular tests. More than 2 g of tissue is needed if low cellular specimens such as muscle, fibrous, and adipose tissue are going to be used (46). During surgery, prolonged anesthesia and ischemic procedures such as arterial ligation may lead to hypoxic tissue damage and decreased pH which subsequently affect the expression level of mRNA causing false low or high quantitation of various mRNAs (12, 49). The age of sample is an important factor that has to be considered in archival samples. Goelz *et al.* reported that DNA fragment sizes extracted from the samples stored for four to six years were often smaller than the DNA sizes of the samples stored less than two years (38). Nevertheless, many laboratories worked with 20-year-old FFPP blocks without any problem (50, 51). Watanabe *et* *al.* evaluated the yield, purity, and integrity of DNA in FFPE tissue specimens. There is a study conducted on Twenty-five lung adenocarcinoma FFPE samples which had been stored at RT for 0.5, 3, 6, 9, and 12 years. They used two methods of DNA extraction and found that the storage time, affects the integrity of DNA but not its absolute yield and purity in FFPE regardless of the extraction method (52). In another study, Wang *et al.* showed a successful amplification from DNA of 40-year-old FFPE tissues but with a declined quantity comparing to fresh lymphocytes (53). The target tissues for molecular analysis usually contain a mixture of various cells and not all of them are the main target for molecular assays. To avoid dilution effect of nucleic acids of undesired cells, marking the area containing the target cells on the hematoxylin and eosin (H&E) slide and corresponding paraffin block is necessary. The next step could be shallow punching on the paraffin block or Laser micro-dissection on the slides. In summary, while blocks stored for years have been used successfully for DNA analysis, it is important to note that the length of amplifiable gene fragments may decrease over time. Potential influences of many factors such as dehydration, clearing, and paraffin reagents, and other conditions on DNA endpoints have remained largely unaddressed in the literature and should be verified in future studies. 

**Non-fixed Tissues**

Care should be taken to maintain the hydration of fresh non-fixed tissues and to avoid drying by covering in gauze soaked with normal saline (46). It is better to place the fresh tissue on wet ice for transportation and to process immediately upon arrival or to keep the tissue in 4ᵒC until extraction. According to the CLSI recommendations, the stability of DNA in tissues stored at 2^o^C to 8^o^C is up to 24 hours, and for those stored at -20^o^C and -70^o^C is at least two weeks and two years, respectively. Room temperature is not suitable even if the tissue is stored for few hours (46, 54). If the tissues are going to be analyzed in the future, they should be frozen in liquid nitrogen or placed in a suitable stabilizing solution as soon as possible to prevent nucleic acid degradation (46). A comprehensive meta-analysis by Greytak *et al.* on case-matched FFPE and fresh or frozen human sample, using the national cancer institute’s Biospecimen Research Database (http://biospecimens.cancer.gov/brd) was done. They revealed that even with variations in analytical parameters, proper method validation, and appropriate handling could increase the correlation between frozen and FFPE tissues (55). Understanding the chemical structure and stability of DNA at RT is also important. Long exposure of DNA to water, reactive oxygen species, and ozone, even in solid state at RT results in DNA degradation and oxidation (56, 57). Some articles demonstrate the advantage of DNA encapsulation by cylindrical glass insertion which is placed in an oxidation-proof metallic capsule for RT storage, in terms of stability and transportation (58). RNA is usually stable in tissue at -70^o^C or -140^o^C (nitrogen vapor) for at least 2 years, but storage at RT, 2^o^C to 8^o^C, or -20^o^C is not recommended (46, 54). RNA storage from Hela cells with encapsulation in RT technology has been developed as an efficient method that provides an anhydrous and anoxic environment (59). 

**Whole Blood, Plasma, Serum, Buffy Coat, and Bone Marrow**

Blood and its components are among the most common samples received in molecular laboratories. Most of the received samples contain anticoagulants, therefore awareness about the possible confounding effects of each anticoagulant and selection of the proper one has paramount importance in ensuring the accuracy of reports. Some anticoagulants used in blood and BM collection, adversely affect the analytical results. There is controversy about the effect of heparin on the molecular assays. There are some reports implying that heparin, if not completely removed during extraction, can inhibit enzyme-based molecular tests (46, 60-64). It has been shown that even low concentrations of heparin may suppress DNA amplification although the inhibition of heparin on PCR using large amounts of DNA may be not significant (64-66). In a disagreeing recent study, it has been shown that heparin is not a significant inhibitor of PCR reaction for intracellular RNA (67). Overall, it is recommended to avoid usage of a heparinized sample as much as possible in molecular assays, but in certain situations such as only one sample or emergency states, a heparinized sample should not be rejected. Cytogenetics studies can be performed on specimens collected in heparin (65, 68). Currently, K2/K3-EDTA and acid citrate dextrose (ACD) are the recommended anticoagulants for molecular assays on blood or BM aspiration samples. Some people suggest that enzyme activity can be inhibited by high levels of sodium EDTA (Royal blue-top tubes for trace element measurements) and should not be used (4, 46, 54). RNA levels can be markedly changed due to degradation by ubiquitous RNases and ongoing production in the cells after sample collection (4, 46). Immediate use of an RNA stabilization solution is strongly recommended. Commercial blood collection tubes containing RNA stabilizers are available to preserve RNA such as PAXgene (PreAnalytiX QIAGEN/BD, Hombrechtikon, Switzerland) and Tempus RNA blood tube (Applied Biosystems Foster City, CA, USA), but their clinical use should be validated (4, 69). 

**Specimen Transport and Storage**

Collected blood can be used directly (whole blood) or be fractionated into serum, plasma, or buffy coat. If the fractions are not intended for short term usage, they should be divided into multiple aliquots into small vials and stored in freezers to avoid multiple freeze-thaw cycles. 

**Whole Blood**

Before DNA extraction, whole blood can be temporarily stored at RT for up to 24 hours or in the refrigerator (2–8°C) for 72 hours (optimum time) (46) or maximum up to 5-6 days. After this time, genomic DNA (gDNA) undergoes remarkable degradation (64, 70). If more delay is unavoidable, the erythrocytes should be removed and the specimen should be frozen at -20ᵒC or -70ᵒC. Exact removal of RBCs is crucial since the heme released during thawing of the frozen sample is an inhibitory agent for PCR (46, 54, 71, 72). Long term stored blood samples in biobanks are increasingly used for a variety of diagnostic/research purposes as a source of genomic DNA. Few studies which are published on this subject imply remarkable results in extracting nucleic acids from whole blood stored in refrigerator or freezer for long durations even up to 30 years (73-75). Whole blood should be collected directly in the tubes containing an RNA stabilizing agent (46, 76). If collecting is not possible, the specimen should be placed on wet ice and immediately transported to the laboratory for extraction of RNA within 4 hours. If RNA extraction is not possible in this period, the samples should be frozen after removing erythrocytes (46, 77). Setting up cold chain transportation or quick addition of RNase inhibitors should be established as an important pre-analytic step for RNA assays. In conclusion, RNA and gene transcription studies should only be performed on samples that are properly collected and stored in RNA stabilizer solutions (46).

**Serum**

Serum is one of the most commonly used samples in clinical laboratories but its use in molecular laboratories is rather limited compared to whole blood and plasma. Although DNA yield of serum may be low, it is reported to be suitable for evaluating genomic DNA (78). Serum should be shipped frozen on dry ice and stored at −20°C prior to DNA or RNA studies (46, 79). DNA is less preserved in serum compared to whole blood (EDTA added) and plasma. To get reliable results, serum should be used in less than one day or in 2-7 days when preserved at RT or at 4°C respectively (80, 81).

**Plasma**

 Obtained data show that DNA levels gradually decrease in a time frame of 9 to 41 months during storage at -80°C (82). The plasma DNA levels gradually decrease over time. This process is delayed by keeping samples in refrigerator. A study showed that storing plasma at 4°C results in a relatively smaller reduction of tumor DNA compared with storage at RT, and the effect is more significant after 96 hours compared to 48 hours (83). Evaluating the effects of different storage temperatures on RNA and DNA levels on unfiltered plasma showed that the storage of specimens in 4°C resulted in stable levels of RNA for up to 24 hours, while DNA levels were stable at both 4°C and RT during 24 hours (84). It is recommended to ship the plasma at 2°C to 8°C and can be kept for up to 5 days. For longer durations, it should be stored at -20°C or lower (46, 77).

**Freeze and Thaw**

The effect of freeze–thaw cycles on nucleic acid quantity and quality is under debate. As a general rule, freezing and thawing have detrimental effects on the concentration of all analytes. Ross *et al.* observed more than 25% decrease in blood DNA concentration after a single freeze–thaw cycle but the quality of DNA did not change even after multiple freeze–thaw cycles (85). According to Chan *et al.*, a single freeze-thaw cycle of plasma has no significant effect on DNA integrity but repeated (3 times) freezing and thawing of plasma (but not extracted DNA) leads to fragmentation of DNA (86). Kopreski *et al.* reported that a single freeze–thaw cycle results in a marked decrease of c-abl and tyrosinase mRNAs in serum (87), however a similar study by Tsui *et al.* showed no significant difference for RNA concentrations between untreated serum/plasma and frozen/thawed samples (84). 

Detection of nucleic acids of infectious agents by molecular methods may be individually considered. According to CLSI, for RNA viruses like human immunodeficiency virus (HIV) (and hepatitis C virus (HCV)), the plasma should be separated from whole blood into a second tube within four hours of specimen collection and WHO/UNAIDS guidelines recommend storing serum and plasma at 4°C –8°C for up to a maximum of one week. For longer storage, the specimens should be frozen at –20°C or lower (46, 88). HIV is a biologically stable virus. It has been stated that HIV-1 RNA is stable in EDTA-whole blood for up to 72 hours and if the plasma is separated by centrifugation within 12 hours the genomic content of the virus can be evaluated for up to 7 days at RT (89). In some studies, HIV-1 RNA levels are found to be stable at RT for up to 30 hours in whole blood and cell-free plasma (90, 91). It has also been demonstrated that storage of EDTA-whole blood and EDTA-cell-free plasma at RT for up to 30 hours, at 4°C for 14 days, and at −70°C for longer durations does not cause significant changes in the viral load (91, 92). HCV RNA is also a stable virus. Comert *et al.* found no significant difference in HCV RNA loads in serum or EDTA-plasma samples stored at RT for 72 hours (93). Jose *et al.* showed that HCV RNA is stable in plasma samples stored at 25°C for 14 days and at 5°C for at least 3 months (94). Hepatitis B virus (HBV) DNA in EDTA-whole blood is stable at RT for 4-6 hours and the serum/plasma should be separated during this time. It can be either kept at RT for 24 hours or preferably be stored at 4°C (ranging from 2°C to 8°C) for seven days (89). Another study corroborated that HBV DNA can be successfully isolated and amplified in samples stored for up to 28 days, both at RT and in the refrigerator, confirming the stable nature of HBV DNA (92). Gessoni *et al.* reported that HBV, HCV, and HIV were stable at 4°C for 72 hours in EDTA anticoagulated whole blood, but HBV DNA showed more stability in this temperature up to 168 hours (95). Viral loads of both HBV DNA and HCV RNA were stable in serum specimens after repeated freezing and thawing (up to 8 cycles) in a survey (96). Salindag *et al.* found that HBV DNA levels in serum specimens remained stable after multiple freeze–thaw (up to 10) cycles (97). 

**Buffy Coat**

Enriched white blood cell (WBC) layer provides a good source of nucleic acids for molecular assays. If there is a delay in DNA extraction within a few days, the buffy coat can be isolated and stored at -70°C (or lower) (98). RNA should be isolated from buffy coat within one to four hours of specimen collection. If not feasible, cells can be kept in RNA stabilizing solutions and stored at RT (46). The duration of storage may differ between various solutions. In patients with eosinophilia, (i.e., chronic myelogenous leukemia) high levels of eosinophil-derived neurotoxin, which has a ribonuclease activity, can decay RNA in buffy coat samples, irrespective of the preservative used (99). 

**Bone Marrow Aspiration**

BM aspirations can be stored at 2°C to 8°C for up to 72 hours upon reception. For longer storage, specimens can be stored at -20°C for several months after the elimination of RBCs. Freezing of specimen without removing RBCs before DNA extraction is not recommended (46, 64). BM aspirations should be put into an RNA stabilization solution as soon as possible, otherwise, the sample should be placed on wet ice without delay and transported to the laboratory. The duration and temperature of storage for each solution may vary and the manufacturers’ instructions should be followed. If no stabilization solution is available and the specimen cannot be frozen, RNA extraction should be done up to four hours after collection (46). If chaotropic agents like guanidium isothyocyanate are added to inactivate RNase, samples can be stored at RT for up to 7 days before testing (64, 100). While some studies have reported acceptable results in RNA quantity of BM samples taken in EDTA tubes and stored at RT for 48 hours, care should be taken in such instances. The type of gene to be tested, the method of RBC removal, and other factors may play a role in obtaining accurate results (101, 102). Unstained excess BM aspirate slides can be fixed in absolute methanol and used for FISH analysis or DNA extraction and subsequent PCR (103). 

**Bronchoalveolar Lavage**

Bronchoalveolar lavage (BAL) can be used to detect microbial agents including mycobacteria, presence of cancerous cells or some genetic diseases such as cystic fibrosis. The source of nucleic acids can be BAL cells such as neutrophils for respiratory syncytial virus (RSV) or cell free BAL fluid for herpes simplex virus (HSV) or cytomegalovirus (CMV) (104). Specimens should be transported and tested within 24 hours of collection (77). If there is a delay more than 24 hours before the assay, specimens should be stored for up to 72 hours at 4°C or longer durations at less than -70°C. For detecting mycobacteria, they should be decontaminated and digested before freezing or long-term storage of the specimens (46, 105). 

**Buccal Cells and Mouthwash Specimens**

These specimens are easily obtainable samples especially for large-scale population-based and epidemiologic studies (106-108). The samples could be obtained by cytobrush, mouthwash, or swabs (106, 109) but mouthwash gives a greater yield and higher quality of human DNA than other methods of collection especially for obtaining high-molecular-weight DNA (108, 109). DNA degradation in specimens collected by cytobrush is higher than mouthwash in RT (109). With long-term storage in RT, the amount of non-human DNA increases due to bacterial overgrowth (109). The alcohol-containing mouthwash specimens are optimal media for preventing bacterial growth on swabs (108). Mouthwash specimen collection is recommended to be done 1 hour after eating or drinking and tooth brushing. The patients should swish the mouthwash several times throughout the mouth for 1 minute and spit it back into the container (106, 110). These specimens are stable at RT for one to two weeks or for at least 6 months if stored at -20°C (106, 107, 110). In cytobrush sampling, patients should not eat or smoke 30 minutes before sampling and should rinse out their mouth with water. The buccal cells should be collected by cotton swabs touching the inner cheek but not the teeth (107). After air-drying for 15 minutes at RT, the swab should be placed in a plastic tube. Foam tipped applicator can also be used for collecting the cells directly from cheeks and transferring the cells to the Flinders Technology Associates (FTA) cards (111, 112). These cards are filter papers that contain chemicals for lysing cells and also stabilizing nucleic acids for long term storage at RT (113). Using cytobrush for exfoliating buccal cells and expectorating the fluid onto special cards containing nuclease-inhibiting and bactericidal material, is another way of sampling (108, 114).

**Cerebrospinal Fluid**

Cerebrospinal fluid (CSF) specimens should be transported at 2 to 8ᵒC for DNA studies. For RNA studies (e.g., enterovirus), they must be immediately put on wet ice and extracted within four hours (77). If extraction cannot be performed in such a short time, RBCs may be removed and the specimen should be immediately frozen and shipped to the laboratory on dry ice. If specimens cannot be processed immediately, they should be placed at -20°C or -70°C or lower (46). According to some authors, DNA in CSF is stable at 22°C to 25°C for up to 24 hours (54), at 2°C to 6°C for 24 to 72 hours (115), at -20°C at least 1 year, and at -70°C more than 1 year (54).

**Pleural, Pericardial, and Other Body Fluids**

Molecular study of body fluids can be used for detection of microbial agents or malignancies. Anticoagulants are not commonly used to collect body fluids. If these specimens are contaminated with red blood cells (115), they are usually pelleted by centrifugation (116). For DNA study, body fluids should be transported to laboratory at 2°C -8°C. For RNA study, they should be transported immediately on wet ice (26). Cell pellets and cell suspensions can be stored at 4°C for brief periods of time (116). RNA extraction should be performed in less than 4 hours after collection (26). For 24-hour storage, cell suspension in a ribonuclease-inhibiting medium is preferable to cell pellets (116). If DNA or RNA extraction cannot be performed in a short time, erythrocytes should be removed before storage at -20°C or less (-70°C is preferred) (26).

**Saliva (Oral Fluid)**

Microorganisms and food remnants in oral cavities contribute to the amount of DNA from saliva samples and may affect the results. For reducing these contaminants, it is recommended that the patients rinse their mouths with water 5 to 30 minutes before saliva collection and avoid eating and drinking after that. It is recommended to collect 2 ml of saliva sample (117). Transport and storage are similar to that of a buccal specimen but if the specimen is scheduled for RNA testing, it should be placed in a transport medium or RNA stabilizing solution or refrigerated at 2^o^C to 8^o^C (118, 119). Oral fluid specimens should be processed within 24 hours of collection (46). Saliva can be quickly used to diagnose congenital CMV infection in newborns. It should be collected by sterile cotton or polyester swab during 21 days after birth, more than one hour after the baby has breastfed. As most CMV seropositive women shed virus in their breast milk, positive CMV PCR results on saliva of infants should be confirmed by urine test (120). 

**Sputum or Gastric Fluid for** ***Mycobacterium tuberculosis***

Sputum specimens should be obtained in a sterile container and then transported at RT for DNA analysis. If transportation time is anticipated to be longer than 30 minutes, samples should be transported to the laboratory at 4 to 8°C. The transport media that have been developed for culture may not be suitable for molecular tests and the media developed for molecular tests are not suitable for culture. The sputum specimens which are not going to be tested immediately should be refrigerated for up to 7 days (121). If longer storage time is needed, the specimen can be stored for few years at -70^o^C (122). For *M. tuberculosis*, it is recommended to first apply N-acetyl-L-cysteine-NaOH for decontamination, then directly put thick particles of sputum on FTA cards by a foam-tipped applicator and spread over an area of 2.5 cm. Subsequently, the samples should be air-dried for 1 hour and finally put in a storage packet and stored at RT (123). Ethanol fixation of sputum samples does not affect the rate of *M. tuberculosis* detection, so the cytology slides of sputum sediments can be used for PCR detection (124, 125). Stomach acidic fluid should be neutralized with phosphate buffer before DNA isolation. After final centrifugation, the pellet can also be used for DNA isolation (126, 127).

**Dried Blood Spot for Viral Genomes**

Dried blood spots (DBSs) can be prepared on filter paper with optimal blood volumes as low as 50 µL. Even six hours lag time between blood collection and its application on DBS can lead to reliable results. Drying procedure takes 3 hours at RT in a safety cabinet (even longer in high humidity) or 1 hour at 37°C in an oven (128, 129). One of the great advantages of DBS is easy handling and shipping even in rural areas with unavailable cold chain (129, 130). RNA in DBS samples is stable at least for 3 months at RT (130). However, some studies show that RNAs are usable as long as 1 year at RT (131, 132). High temperature (37°C) results in progressive degradation of RNA but has less effect on DNA. RNA progressive loss at 37°C is remarkable after two months (128). Refrigerated (in a zip bag with desiccant pack) and frozen DBS samples can be stored for 1 year and a period of 4 years, respectively, with reliable recovery of HIV1 RNA (133). 

**Stool**

Stool sample preservation in cold condition (4°C) is recommended during transportation. Allowable transportation time at 4°C is 24-48 hours (134-136). This time is 4 hours at RT without preservation (137, 138). Fixatives/preservatives such as TotalFix, Unifix, Zinc- or copper- based polyvinyl alcohol (PVA), and Ecofix can preserve specimens for storing and transporting at RT (139). DNA stabilizer can be utilized for sample preservation to protect DNA and/or RNA and prevent degradation after collection (140, 141). One of the most famous stabilizers is RNAlater, which prevents RNA as well as DNA degradation at ambient temperature for one week. Another common and economic stabilizer for fecal DNA is 95% Ethanol (134, 141-143). The procedure contains mixing 2.5 mL of 95% ethanol with approximately 1–2 g of stool (144). For microbiome studies, it is ideal to transport fecal specimens immediately at -20°C or -80°C. These specimens can be stored at -80°C for up to 2 years with unremarkable alteration in stool microbial composition. The storage time is reduced to few weeks at -20°C (141, 145). 

**Urine**

Urine samples can be used for molecular evaluation of kidney, bladder and prostate neoplasia, genetic anomalies, and a number of infections in these organs (146). Immediate analysis is ideal, but it may not be usually achievable, so storage and transportation of urine need particular attention to prevent nucleic acid degradations (146, 147). It is better to avoid storing fresh unprocessed urine samples in ambient temperature as much as possible (148). In a study in Italian patients, adding EDTA with 40 mM final concentration has been recommended to preserve human DNA in urine for up to 28 days at RT, 4°C, and -80°C. Human DNA stability is different according to geographic origin due to changes in urine matrix (146). Collecting second-morning urine specimen is advocated and addition of EDTA and storing urine supernatant at -20°C are useful for using urine cell free DNA (ucfDNA), (149). Urinary methylated DNA can be used to detect bladder and prostate cancers (150). Adding EDTA can lead to more reliable PCR results for evaluation of methylated DNA (147). Addition of PenStrep with no negative effect on DNA preservation has to be considered to prevent bacterial overgrowth (147). Evaluation of *Chlamydia trachomatis* DNA storage at RT for 1 week without preservation (151) and *Trichomonas vaginalis* DNA at 4°C for 30 days with EDTA containing kit (urine preservative transport (UPT)) are possible (152). Urine miRNAs are more stable and nuclease resistant compared to large mRNA and snRNA (small nuclear RNA) (153), and other cellular RNAs (154, 155). There was no significant difference in the yield of miRNA between fresh urine samples and frozen urine samples after 90 days (153). It is mentioned that no significant difference exists between miRNA levels in urines regarding storage at RT up to 72 hours or after seven cycles of freezing and thawing (156). In another study, 23–37% of the initial amount of miRNAs in urine samples are stable at RT in 5 days (157). Storing urine sample at 4°C helps preservation of miRNA for 5 days (157). Urinary cell-free RNA (cf-RNA) measurement can be used for detection of renal cell carcinoma. Cell-free urine is obtained after two steps of centrifugation at 4°C and can be stored at RT or 4°C for 24 hours. Freezing in liquid nitrogen and thawing don’t change the concentration of RNA (158). Viral infections can be detected in urine using PCR, using sterile containers for urine sample collection and transport at 4°C (159). Urine can be stored at 4°C for Epstein–Barr virus (EBV) and CMV until processing is done in one week (160, 161). The specimen should be shipped on wet ice within one week for CMV evaluation (160). Collection of the first 10-20 mL of voided urine for HSV (first catch) and transportation within 3 hours has been recommended. The specimens can be kept at -70°C for up to 3 months, but individual manufacturer’s instructions should also be considered for storage conditions (162). 

**Buccal and Nasopharyngeal Swabs**

Collection of buccal cells by swab is becoming a widely used procedure for genotyping, detection of early signs of cancer, and regional infection diagnosis (163-166). For DNA testing, buccal (or epithelial) cells could be collected and transported at RT. They are stable for approximately one week (167, 168). For RNA testing, samples must be collected and transported in a suitable RNA stabilizing agent (169). For suspected mumps parotitis, the buccal swab should be placed into viral transport medium (VTM) by rubbing the swab against rim of the tube and can be kept for at least 1 hour at 4°C. It should be transported on cold packs at 4°C within 24 hours. If there is an over 24 hours hesitation in delivery, specimens should be frozen at -70°C (166). Nasopharyngeal specimens for Respiratory viruses diagnosis (Influenza, Respiratory Syncytial Virus, Parainfluenza virus, Human Metapneumovirus, Rhinovirus, Enterovirus, Adenovirus, Coronavirus) are collected by sterile dacron/nylon swab (170, 171). Nylon flocked swabs are more efficient in comparison with other synthetic ones like rayon swabs (172, 173). The most common acceptable specimen for detecting Influenza Viruses is nasopharyngeal swabs along with washes or aspirates. Throat swabs and/or nasal swabs (two sterile dry polyester swabs with aluminum or plastic shafts) are also acceptable (134, 174). However, more viral loads can be obtained by nasopharyngeal swabs in comparison with oropharyngeal swabs (172). The swab should be transported into sterile VTM at 4°C. If the shipment is delayed by 3 to 4 days, the samples should be kept at -70°C (134). 

**Cervical and Urethral Swabs**

Detection of sexually transmitted diseases (STDs) has been revolutionized due to molecular techniques progression (175). Screening Human papillomavirus (HPV) DNA is a useful cancer screening method to prevent cervix cancer (177). Importantly, the results of clinical tests are highly dependent on appropriate shipping on wet ice, and storage of specimens at 2°C to 8°C in the laboratory within 7 days of collection (176). Swabs can be transported dry inside a sealed tube or placed in transport mediums. DNA stability can be maintained at 2°C to 8°C for up to 10 days. Resuspension of swabs in transportation fluid can be done according to the manufacturer’s instructions and stored at -70°C or a lower temperature. Immediate centrifugation and processing of pellets of DNA and RNA can also be done. Frozen specimens can be thawed and processed similar to fresh samples (46). Dry vaginal swab shipping and wet transportation in liquid medium are accurate for *C. trachomatis* and *Neisseria gonorrhea* (175), and the accuracy of dry and wet shipped intravaginal swabs for PCR evaluation of *C. trachomatis* and *N. gonorrhea* is the same (175), but few specialized transport media are recommended by some authors (176). In addition, cervical specimens for HPV collected by swabs can be stored up to 1 month at ambient temperature (177). Again, for *C. trachomatis*, *Hemophilus ducreyi*, *Klebsiella granulomatis*, *N. gonorrhea*, *Mycoplasma genitalium*, *Treponema pallidum*,* T. vaginalis*, dry shipping or VTM at RT can be used within 1 day or at 4°C up to 4 days (89). Furthermore, HSV, HPV (human papilloma virus) and Adenovirus swabs, can be dryly transported or VTM can be used in RT within 1 day or at 4°C for up to 4 days (89). 

**Prenatal Molecular Screening**

Prenatal testing aims to help families make health care decisions before and after giving birth or decide to terminate pregnancy. Prenatal specimens include samples with fetal or placental origin, collected before birth. Chorionic villus sampling (CVS), amniotic fluid (AF), and maternal plasma are the most referred samples and the most common reason for CVS or amniocentesis is to request for cytogenetic studies such as karyotype and FISH. Interpretation of the results from prenatal diagnosis (PND) testing contributes to critical decisions by the clinicians and parents such as terminating a pregnancy so that diagnostic errors can lead to serious consequences such as legal issues and a heavy emotional and financial burden on the family and the society especially when the error results in the birth of an affected child (178-180). Some specific general considerations should be taken into account when dealing with PND specimens in the clinical laboratory: 

1. The laboratory should make sure that the patient has gone through proper consultation sessions and informed about the accuracy of the tests and potential treatments for the fetus. Valid and informative written consent must be taken (181-185).

2. Maternal cell contamination (MCC) in chorionic villus or AF samples is a significant pre-analytical source of errors (186). The possibility of MCC should always be considered while dealing with fetal samples and maternal blood should be sent with fetal specimen (AF/CVS) to exclude MCC (182, 186). 

3. Prenatal diagnosis specimens must be labeled with mother's full name, type of specimen, gamete donation, and surrogate pregnancy if applicable (181, 182). 

4. In the case of bearing twins or multiple pregnancies, accurate labeling of the specimens is of critical importance. The rules of standard practice should be observed to guarantee that samples are obtained from different fetuses and correctly labeled (181).

5. If linkage analysis is needed, DNA from parents and an affected family member should be analyzed together with the fetal sample (187).

6. Because of the time and legal restraints in pregnancy, the prenatal sample is usually immediately processed or transported. The remaining DNA sample is stored according to the previously mentioned standards (46, 183, 188).

**Chorionic Villus Sampling Specimens**

The chorionic tissue should be collected in normal saline with 10 mM EDTA and separated from maternal tissue under a stereo-microscope or dissecting microscope to evaluate contamination with endometrial decidual tissue of maternal origin (189, 190). Alongside the CVS samples, maternal blood sample should be sent to assess MCC. The decidua should be removed carefully before shipping or processing and CVS specimens devoid of MCC should be processed on the same day of arrival at the laboratory. At least 15 mg of a CVS specimen (after maternal tissue removal) in sterile tissue culture medium or saline buffer is recommended for shipping at ambient temperature (46). Association for Molecular Pathology requires 5 mg to 10 mg of cleaned, isolated CVS tissue (186). Placental mosaicism errors could be prevented if at least two CVS samples are taken from different sites of the placenta (191). 

**Amniotic Fluid**

For amniocentesis, a needle is inserted into the amniotic sac and an amniotic fluid sample is obtained. Subsequently, 15 to 20 ml of the amniotic fluid is placed into a sterile container (181). Between 0.5 to 4 ml or 1/10 of the sample is usually taken for quantitative fluorescent PCR (QF-PCR); meanwhile, the remainder is preserved for karyotype analysis (191). In the case of receiving a blood-tinged specimen, documentation is necessary. Maternal blood (4 ml in EDTA tube) is also collected to examine maternal contamination. Amniotic fluid is transported to laboratory at ambient temperature. If DNA could not be extracted on the arrival day, it should be stored at 2°C to 8°C to be extracted on the next day. A backup culture is required for possible additional tests. Amniotic fluid can be processed without culture after 15 weeks of gestation (46, 181). According to CLSI, the optimum volume of amniotic fluid is at least 10 ml. In the guideline issued by the Association for Molecular Pathology, the median quantity of material requested for direct AF testing is said to be 12 ml (46, 182). Maternal cell contamination is correlated with the amount of blood present in the amniotic fluid which could be assessed by examining the blood pellet after centrifugation (192). Extracted DNA from CVS or amniotic fluid cultures could be used as material for molecular testing if it is not done later than 2 weeks after the cytogenetic report date. In many laboratories, it is a common practice to maintain the cell culture as a backup until results are reported (181, 191). 

**Circulating Cell-Free Fetal DNA in Maternal Plasma**

Fetal molecular and cytogenetic abnormalities are detectable by analysis of circulating cell-free fetal DNA (ccffDNA) from the maternal plasma. Fetal fractions (FF) usually range from 2% to 40% with a mean of 10% of total circulating cell-free DNA (ccfDNA) in maternal blood across varying gestational ages (193-195). The lower limit of ccfDNA for an accurate result is about 4% (196). At FF quantities lower than 4%, no result should be reported. Maternal blood should be collected in standard EDTA tubes and plasma preparation should be performed within 6 hours, which starts with a low-speed centrifugation. The removed plasma is then centrifuged at a higher speed to separate any residual debris as sediment. Whole blood can be shipped at ambient temperatures (6–37°C). Several companies have introduced reagents that prevent white blood cell degradation and impede maternal DNA release (194). 

**Table 1:** Preanalytical Recommendations for Molecular Analysis

Specimen type	Target	Temperature	Duration	Reference
Whole blood	DNA	RT	up to 24h	(46)
		2-8°C	up to 72h optimal, but possible up to 6 days	(46) (70)
Whole blood	DNA (HBV)	RT	4-6h	(89)
	RNA (HIV, HCV)	4°C	72h	(95)
Serum	DNA	RT	24h	(89)
	DNA (CMV)	RT	less than one day	(80)
	DNA (CMV)	4°C	2 days	(81)
	DNA (HBV)	RT	24h	(89)
	DNA (HBV)	4°C	7 days	(89)
Plasma	DNA	RT	24h	(84)
		2-8°C	5 days	(46,77)
		-20 °C	longer than 5 days	(46,77)
		-80°C	9 to 41 months	(82)
Plasma	RNA	4°C	up to 24h	(84)
Plasma	DNA (HBV)	RT	24 h,28 days	(89,94)
		4°C	7 days, 28 days	(89,94)
Plasma	RNA (HIV, HCV)	4–8°C	1 week	(88)
	HIV	RT	30h	(90)
	HIV	RT	7 days	(91)
	HIV	5°C	14 days	(89)
	HCV	RT	72h	(92)
	HCV	25°C	14 days	(93)
	HCV	RT	3 months	(94)
Dried blood spot	RNA	RT	up to 3 months	(131,132)
		4°C	up to 1 year	(133)
		-20°C	up to 4 years	(133)
Stool	DNA	RT	4h	(137,138)
		4°C	24-48h	(134,136)
		-20°C	few weeks	(141,145)
		-80°C	2 years	(141,145)
Nasopharyngeal swabs (in VTM)	Respiratory viruses	4°C	3-4 days	(166)
		-70°C	more than 3-4 days	(166)
Cervical swab	DNA	2–8°C	10 days	(46)
	DNA (Neisseria gonorrhea, Chlamydia trachomatis)	2- 8°C	up to 7 days	(176)
	DNA (HPV)	RT	1 month	(177)
	DNA (Hemophilus ducreyi, Klebsiella granulomatis, Mycoplasma genitalium, Treponema Pallidum, Trichomonas vaginalis)	RT	1 day	(89)
		4°C	4 days	
Amniotic fluid	DNA	2-8°C	24h	(46)

Abbreviations: CMV: cytomegalovirus, HBV: hepatitis B virus, HCV: hepatitis C virus, HIV: human immunodeficiency virus, RT: room temperature, VTM: viral transport medium

##  Conclusion

It is acknowledged that implementation of the recommendations presented herein, may require changes in workflows, schedules, or materials. However, it is also notified that one of the important prerequisites of this implementation is reliable documentation of the pre-analytical history of patients’ specimens which enables objective clues in getting false positive or negative data. In translational research laboratories, specimen pre-analytical variables are both uncontrolled and undocumented. In addition, these laboratories do not routinely perform clinically mandated quality control that may reveal poor specimen quality before testing and the generated data may be completely uninterpretable or inconclusive (3). Therefore, coming to an agreement regarding the specific variables in patient specimen acquisition, handling, processing, storage, and transportation causing most of the quality compromise and molecular alterations, would ensure that the research data have enough reliability as well as reproducibility to foster translational process from bench to bedside.
